# Distinct Mechanisms for Increased Cardiac Contraction Through Selective Alteration of Either Myosin or Troponin Activity

**DOI:** 10.1016/j.jacbts.2022.04.013

**Published:** 2022-09-07

**Authors:** Rohit R. Singh, Rebecca E. Slater, Jinghong Wang, Chen Wang, Qi Guo, Alykhan S. Motani, James J. Hartman, Sakthivel Sadayappan, Brandon L. Ason

**Affiliations:** a Amgen Research, Department of Cardiometabolic Disorders, Amgen, South San Francisco, California, USA; b Heart, Lung and Vascular Institute, Department of Internal Medicine, Division of Cardiovascular Health and Disease, University of Cincinnati, Cincinnati, Ohio, USA; c Discovery Biology, Cytokinetics, South San Francisco, California, USA

**Keywords:** cMyBP-C phosphorylation, heart failure, MYBPC3, myosin, troponin, AAA, transgenic mice expressing alanine at Serine 273, 282, and 302, cMyBP-C, *c*ardiac myosin binding protein-C, DDD, transgenic mice expressing aspartate at Serine 273, 282, and 302, F_max_, maximal force per cross-sectional area, HF, heart failure, KO, knock-out, k_tr_, rate of force redevelopment, LV, left ventricle, M, myosin-selective small molecule, OM, omecamtiv mecarbil, pCa_50_, half maximal effective calcium concentration, SRX, super relaxed state, T, troponin-selective small molecule, tWt, transgenic wild type

## Abstract

Modulation of sarcomere contractility represents a new therapeutic opportunity for the treatment of heart failure by directly targeting the thick and thin filament proteins of the sarcomere to increase cardiac muscle contraction. This study compared the effect of 2 small molecules (M and T) that selectively alter myosin thick filament (M) or troponin thin filament (T) activity on overall cardiac muscle mechanics. This study revealed key differences related to the mechanism utilized by M and T to increase contractile force generation and suggests that targeting different proteins within the sarcomere may result in differentiating therapeutic profiles.

## Highlights

• Myosin-based thick filament (CK-138 or M) and troponin-based thin filament (CK-030 or T) selective compounds improved cardiac muscle contraction and calcium sensitivity
• T exhibited faster tension redevelopment (*k*_*tr*_) and ATP exchange rates relative to M.
• Importantly, phosphorylation status of cardiac myosin binding protein-C did not alter M and T activity.

## Visual Abstract

**Figure:**
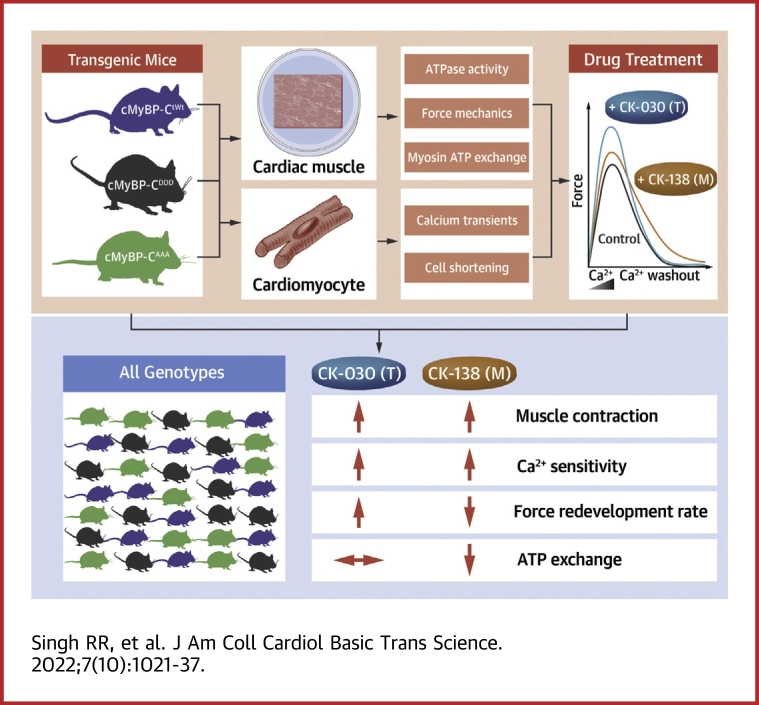

Heart failure (HF) is a leading cause of death worldwide, with more than 200,000 cases annually within the United States alone.^1^ It affects 2% of the population within industrialized nations, climbing to 10% for individuals who are 65 years of age or older.^2^, ^3^, ^4^ HF prevalence leads to more than 1 million hospitalizations per year, and medical costs are anticipated to increase significantly by 2040.^5^^,^^6^

Molecules that directly target sarcomere protein activity represent a new therapeutic class for the treatment of HF that aim to improve cardiac muscle contraction. Sarcomeres, the basic functional unit of striated and cardiac muscle, facilitate muscle contraction through the movement of the actin thin filament over the myosin thick filament. This process is coupled to myosin ATPase activity and regulated both by changes in intracellular calcium concentrations and by post-translational modifications to the sarcomere proteins.^7^, ^8^, ^9^, ^10^, ^11^

Omecamtiv mecarbil (OM) improves contractile performance by stimulating myosin.^12^, ^13^, ^14^ OM increases myosin ATPase activity, allowing for increased contractile performance in cardiac muscle.^13^^,^^14^ In the COSMIC-HF (Chronic Oral Study of Myosin Activation to Increase Contractility in Heart Failure) phase II and the GALACTIC-HF (Registrational Study With Omecamtiv Mercabil [AMG 423] to Treat Chronic Heart Failure With Reduced Ejection Fraction) phase III clinical trials, OM was shown to reduce left ventricular (LV) diameter and improve cardiac function, and resulted in a modest but significant reduction in the time to first event in the composite endpoint of cardiovascular death or HF events, respectively.^15^, ^16^, ^17^, ^18^ HF is a heterogenous disease, and efforts are currently underway to better understand if a subgroup of patients within GALACTIC-HF exhibited a greater therapeutic benefit with OM to identify those best suited to receive this medicine.^15^

Several post-translational modifications to the sarcomere resulting in abnormal contractile performance are associated with HF.^19^^,^^20^ These modifications impede cross-bridge cycling, the process by which myosin binds to and releases actin during muscle contraction. Cardiac myosin binding protein-C (cMyBP-C) is a sarcomere protein that has multiple functional roles that influence contraction. When dephosphorylated, it promotes the super relaxed state (SRX), or less active state of myosin; however, when phosphorylated, it facilitates sarcomere contraction by activating actin thin filaments.^21^ Its phosphorylation is essential for normal cardiac function.^9^^,^^22^, ^23^, ^24^, ^25^ cMyBP-C consists of 8 immunoglobulin- and 3 fibronectin-like domains, C0-C10, and is found in repeating stripes throughout the C-zone of the sarcomere.^26^ The importance of cMyBP-C is highlighted by the fact that allelic variants in cMyBP-C are highly prevalent in hypertrophic cardiomyopathy and implicated to an extent in dilated cardiomyopathy.^27^, ^28^, ^29^, ^30^ The phosphorylation of serine residues at 273, 282, and 302 within the m-domain of cMyBP-C are essential for normal cardiac function.^26^^,^^31^^,^^32^ cMyBP-C is 50% dephosphorylated in failing hearts regardless of disease etiology.^33^, ^34^, ^35^ Additionally, the ablation of cMyBP-C phosphorylation leads to pathological hypertrophy in animal models.^36^ The phosphorylation of cMyBP-C destabilizes SRX, a myosin head configuration that is inactive and not actively undergoing cross-bridge cycling. Phosphorylation of cMyBP-C therefore results in increased contractility by decreasing the proportion of myosin heads in SRX, whereas dephosphorylation has a stabilizing effect on SRX, resulting in decreased contractility.^37^ Given the impact this post-translational modification has on cross-bridge cycling and HF, questions remain regarding how this modification may influence the overall effectiveness of small molecules that target sarcomere proteins to increase cardiac contraction.

Here, we used a transgenic mouse model in which phosphorylation sites at serines 273, 282, and 302 within the m-domain of cMyBP-C were replaced with the phospho-null residue alanine (AAA).^36^^,^^38^^,^^39^ The AAA transgenic mice expressed 40% cMyBP-C^AAA^ and displayed no changes in morbidity or mortality, but displayed depressed cardiac contractility, altered sarcomere structure, and upregulation of transcripts associated with a hypertrophic response.^40^ Transgenic mice in which the phosphorylation sites were replaced with a phospho-mimetic residue, aspartate (DDD), had no phenotype, consistent with the observation that ∼90% of cMyBP-C was at least mono-phosphorylated in healthy hearts.^26^^,^^33^ Transgenic mice in which the AAA or DDD allelic variants were crossed into a cMyBP-C knock-out (KO) background (AAA[KO] and DDD[KO]) resulting in 100% mutant protein replacement have been extensively characterized.^26^^,^^38^^,^^41^^,^^42^ However, the phenotype of AAA(KO) mice was quite severe, exhibiting overt hypertrophy and myocyte disarray.^36^^,^^38^ We found the AAA(WT) transgenic line to be a more interesting model for this study both for the milder phenotype as well as the observation that failing human hearts exhibited ∼50% reduced, but not fully ablated, phosphorylation of cMyBP-C.^31^^,^^33^^,^^40^^,^^43^

The study aims were to compare the effects of a myosin selective small molecule that is an OM surrogate, CK-1317138 (CK-138 or M), with a troponin selective small molecule, CK-3826030 (CK-030 or T), on contractile mechanics, as well as to further investigate the impact that cMyBP-C phosphorylation has on their activity. To answer these questions, a series of experiments with M and T were performed using cardiac tissue and cells isolated from all 3 transgenic lines. The study demonstrated that both myosin- and troponin-selective compounds were able to improve contractile function irrespective of the cMyBP-C phosphorylation state. This study additionally revealed differences between M and T, where T leads to greater force redevelopment and ATP exchange rates.

## Methods

### Mouse models

The transgenic mice used for the experiments were derived from wild-type mice in the FvB/N background. The 3 serine residues (ser 273, ser 282, and ser 302) in the m-domain were replaced by alanine residues for the AAA cMyBP-C hypo-phospho-mimetic transgenic ^36^ and were replaced by aspartate residues for the DDD cMyBP-C hosphor-mimetic transgenic.^26^ These mice were compared with the transgenic overexpression of wild-type cMyBP-C (tWt). All in-life animal care and handling were in accordance with IACUC guidelines at either University of Cincinnati or Amgen.

### Skinned papillary fiber force mechanics

Papillary fibers were carefully dissected from the left ventricles of 12-14 weeks old FVB/N mice of all 3 genotypes (tWt, AAA, and DDD).^32^^,^^44^ Papillary fibers were skinned over night at 4°C in 1% Triton X-100 in relaxing buffer (55.74 mmol/L potassium propionate, 7 mmol/L ethylene glycol bis(2-aminoethyl) tetraacetic acid, 100 mmol/L N,N-bis(2-hydroxyethyl)-2-amino ethanesulfonic acid, 0.02 mmol/L calcium chloride, 5.5 mmol/L magnesium chloride, 5 mmol/L dithithreitol, 15 mmol/L creatine phosphate, and 4.7 mmol/L adenosine triphosphate with pH adjusted to 7.0 with 4 mol/L potassium hydroxide and ionic strength maintained at 180 with potassium propionate) with pCa 9.0. Skinned papillary fibers were sequentially exposed to increasing calcium solutions with pCa (6.3, 6.0, 5.8, 5.7, 5.6, 5.4, and 4.5). The different pCa solutions were made by mixing relaxing buffer pCa 9.0 and activating solution pCa 4.5. Maximal calcium solution (pCa 4.5) had a similar chemical makeup as relaxing buffer except it had 7 mmol/L calcium chloride. For the measurement of maximal force and calcium sensitivity, a single papillary fiber was cycled through all of the pCas 3 times, first with no compound added followed by increasing (1× and 3×) concentrations of the experimental compounds. For the 1× and 3× compound treatment, the skinned papillary fibers were incubated for 300 seconds in pCa 9.0 with either 1× or 3× compound followed by cycling it through all of the pCas containing the compound dosage. Rate of force (tension) redevelopment (*k*_tr_) was measured (Aurora Scientific Inc) as previously described.^45^ Briefly, once the fiber reached a steady state force in submaximal pCa of 5.7 it was shortened by 20% for 20 milliseconds before a rapid (∼1 millisecond) stretch back to its original length. *k*_tr_ was calculated by fitting the force redeveloped in the fiber, after this slack-stretch treatment against the time required to one-phase association curve.^45^

### Nucleotide chase experiments

The nucleotide chase experiments were performed as described previously.^46^^,^^47^ In short, a small section of cryopreserved left ventricle from mouse hearts were incubated in skinning buffer (NaCl, 100 mmol/L; MgCl_2_, 8 mmol/L; EGTA, 5 mmol/L; K_2_HPO_4_, 5 mmol/L; KH_2_PO_4_, 5 mmol/L; NaN_3_, 3 mmol/L; ATP, 5 mmol/L; DTT, 1 mmol/L; BDM, 20 mmol/L; Triton-X 100, 0.1%, pH 7.0) for 6 hours at 4°C. The skinning buffer was changed every 2 hours. After 6 hours, skinned fibers were then immersed in glycerinating buffer (K acetate, 120 mmol/L; Mg acetate, 5 mmol/L; K_2_HPO_4_, 2.5 mmol/L; KH_2_PO_4_, 2.5 mmol/L; MOPS, 50 mmol/L; ATP, 5 mmol/L; BDM, 20 mmol/L; DTT, 2 mmol/L; glycerol, 50% [v/v], pH = 6.8) overnight at 4°C under gentle agitation. For the mANT-ATP turnover experiments, a small, glycerinated fiber (∼800 μm × 150 μm) was placed on a cooled slide. The fiber was fixed onto the slide by double-sided tape to create a chamber using a glass cover slip. The glycerinated fiber was washed with rigor buffer (K acetate, 120 mmol/L; Mg acetate, 5 mmol/L; K_2_HPO_4_, 2.5 mmol/L; KH_2_PO_4_, 2.5 mmol/L; MOPS, 50 mmol/L; DTT, 2 mmol/L at pH = 6.8) 5 times, followed by a final wash by rigor buffer with 250 μmol/L of mANT-ATP. The fibers were incubated in the presence of mANT-ATP for 5 minutes. Later, the slide was placed in the microscope chamber, and the 10× objective of Leica DMi8 microscope was used to image the slide. The slide was imaged every 5 seconds for 600 seconds by exciting the slide at 395 nmol/L by DAPI filter cube with exposure of 20 milliseconds to avoid photobleaching. After 60 seconds, the mANT-ATP buffer was washed away with ATP-chase buffer (K acetate, 120 mmol/L; Mg acetate, 5 mmol/L; K_2_HPO_4_, 2.5 mmol/L; KH_2_PO_4_, 2.5 mmol/L; MOPS, 50 mmol/L; ATP, 4 mmol/L; DTT, 2 mmol/L at pH 6.8) to allow the myosin in the fiber to exchange the fluorescent mANT-ATP with regular nonfluorescent ATP. For M and T, the glycerinated fibers were treated with the rigor buffer, mANT-ATP buffer and ATP-chase buffer containing the indicated concentration of the compound.

The decay in fluorescence was calculated by subtracting the background fluorescence from the fluorescence of the fiber at 3 different regions of the fiber. The decay in fluorescence was fitted to a custom defined 2-state exponential decay curve to yield the biphasic exchange of mANT-ATP with ATP in accordance with decrease in fluorescence:

$$I=1-F\left(1-\exp\left(\frac{-t}{T_{F}}\right)\right)-S\left(1-\exp\left(\frac{-t}{T_{S}}\right)\right)$$

where I = observed fluorescence intensity, F and S = percentage proportion of fluorescence decay for rapid or fast exchange (F) and slow exchange (S) of mANT-ATP with ATP, respectively, and T_F_ and T_S_ = time constants for the lifetime of the 2 phase mANT-ATP exchange.

### Cardiomyocyte contractility measurements

Left ventricular cardiomyocytes were isolated from cMyBP-C AAA, cMyBP-C DDD, and the control cMyBP-C transgenic tWt mice. Following heart excision, the aorta was cannulated on a Langendorff apparatus (Harvard Apparatus). The heart was perfused retrograde with Tyrode buffer (in mmol/L: 140 NaCl, 5.4 KCl, 0.33 NaH2PO4, 0.5 MgCl_2_, 5 HEPES, and 11 D-glucose, pH 7.4) at 2.5 ml/min, 37°C for 4 minutes. The heart was then perfused with enzyme solution using the adult mouse cardiomyocyte isolation kit following the manufacturer’s instructions (Cellutron Life Technologies, ac-7034). After digestion, the heart was removed from the cannula and the left ventricle was gently teased apart. Cell suspensions were obtained by gentle pipetting. After spinning, cardiomyocytes were transferred to Tyrode buffer with Ca^2+^.

Cardiomyocyte contractility was measured using an IonOptix contractility system. Myocytes were loaded in the perfusion chamber of the microscope stage filled with perfusion buffer (Tyrode buffer with 1.8 mmol/L CaCl_2_, 0.01% DMSO). After stabilization, the myocytes were perfused with perfusion buffer at room temperature. They were field stimulated with 12 V at 1.0 Hz with a 1.0 milliseconds pulse width using a pair of platinum wires placed on opposite sides of the chamber connected to a MyoPacer stimulator. Myocytes perfused with perfusion buffer were recorded at baseline, then perfused with compound for 3-4 minutes. Analysis was performed using the IonWizard software (IonOptix). For each test, an average of 10-15 sarcomere shortening traces was analyzed. Cells were selected that were beating and appeared grossly normal. Cells were not considered for recording if they were not contracting or, in very rare instances, appeared to be exhibiting an abnormally large reduction in sarcomere length (an abnormally large contraction). All recorded cells were analyzed. Cells with poor data quality were excluded. The remaining cells were recorded and exhibited a 7%-15% reduction in sarcomere length relative to its resting sarcomere length at baseline before treatments were given. Cells were isolated from 2 to 4 animals for each group. Neither the genotype nor the treatment disproportionately influenced cell exclusion.

### Myofibril ATPase experiments

Mouse myofibril preparation: 35 to 50 frozen mouse hearts were defrosted on ice. The tissue was chopped in a Waring blender with cold buffer A (25 mmol/L PIPES, 50 mmol/L KCl, 5.0 mmol/L MgCl_2_, 1.0 mmol/L NaN3, and 1.0 mmol/L DTT, pH 7.0) and then homogenized with 1300D Polytron Homogenizer (12-mm generator) for 2 minutes at 30,000 revolutions/min on ice. The homogenization step was repeated 3 times. The homogenate was centrifuged at 5,000 revolutions/min for 10-15 minutes. After centrifugation, all the supernatants were discarded, and a light brown layer of mitochondria, sarcolemma, and sarcoplasmic reticulum on top of the pellet was scraped off. The pellet was homogenized with buffer B (25 mmol/L PIPES, 50 mmol/L KCl, 5.0 mmol/L MgCl_2_, 1.0 mmol/L NaN3, 1.0 mmol/L DTT, and 2 mmol/L EGTA, pH 7.0). The suspension was then centrifuged at 9,000 rpm for 15 minutes. The supernatant was discarded, and a light brown layer on top of the pellet was scraped off. The pellet was homogenized with buffer C containing 1% Triton X-100 (25 mmol/L PIPES, 50 mmol/L KCl, 5.0 mmol/L MgCl_2_, 1.0 mmol/L NaN3, 1.0 mmol/L DTT, 2 mmol/L EGTA, and 1% Triton X-100, pH 7.0) to remove mitochondrial, sarcolemmal, and sarcoplasmic reticular membranes. After the pellet was fully resuspended in buffer C, the suspension was then centrifuged at 4,200 revolutions/min for 10 minutes. This sequence of resuspension, homogenization, and centrifugation was repeated another 2 times. The resulting light brown pellet was filtered through Nitex nylon mesh, 630 MICRON (Genesee Scientific Corp, catalog number NC9432449), to remove any residue of coarse tissue. Then, the previous sequence of resuspension, homogenization, and centrifugation was repeated another 2 times. Finally, Triton X-100 was removed with buffer A wash 3 times. In each wash, the pellet was homogenized in Buffer A, and the suspension was centrifuged at 4,200 revolutions/min for 10 minutes. All supernatants were discarded, and the top light brown layer of each pellet was removed. After 3 washes, the pellet was homogenized in buffer A. The homogenate obtained after 3 washes was white in color. The homogenate was then aliquoted and stored at −80°C.

### Myofibril assay procedure

The experiment was performed in an assay buffer containing 15 mmol/L PIPES (pH 7.0), 5.0 mmol/L MgCl_2_, 10 mmol/L KCl, and 1.0 mmol/L freshly added DTT. All reagents were prepared with the assay buffer. The 12 mmol/L CaCl_2_ solution and 5.0 mmol/L EGTA solution were dispensed at 5.0 and 10 μL, respectively, into a 384-well black plate with a clear bottom. The compound solution to be titrated was prepared as a 10 mmol/L stock in DMSO, and serially diluted (1:3) over 7 points, and the concentration of compound in the last point was 0. The compound solution at each concentration was dispensed at 0.50 μL into the plate. Myofibrils were fully thawed on ice and homogenized with 1300D Polytron Homogenizer for 30 seconds at 20,000 revolutions/min. Then, the homogenized myofibrils were diluted to the desired concentration and 10 μL myofibril solution was dispensed into the plate. A reaction solution containing 4.0 mmol/L ATP, 1.6 mmol/L NADH, 3.0 mmol/L phosphoenolpyruvate, 30 U/mL lactate dehydrogenase, and 30 U/mL pyruvate kinase was freshly prepared and dispensed at 25 μL into the plate to initiate the myofibril-catalyzed reaction. Upon finishing dispensing, the 384-well plate was kinetically read at 340 nm at every 30 seconds for 1 hour.

### Reconstituted sarcomere myosin ATPase assay

The myosin ATPase activity in a reconstituted sarcomere were performed based on the previous study.^48^ In short, myosin,^49^ tropomyosin,^50^ actin,^51^ and troponin complex^52^ were isolated and purified from bovine left ventricle (cardiac), rabbit back muscle (fast skeletal), and bovine masseter tissue (slow skeletal). All muscle tissues were obtained from Pel-Freez Biologicals. The purified myosin was further subjected to α-chymotrypsin digestion to yield soluble myosin S1 heads.^49^ The active myosin S1 heads were purified by adding equimolar ATP and F-actin to soluble myosin S1 heads, further centrifuging the dead myosin S1-actin complex at 10,000 revolutions/min in 4°C.^53^ The reconstituted sarcomere consisted of myosin S1 of 1 muscle origin and coupled with thin filament (actin, troponin, and tropomyosin) of same or other muscle origin in PM12 buffer made of 1 mmol/L ATP, 12 mmol/L Pipes, 2 mmol/L MgCl_2_, and 1 mmol/L DTT, pH 6.8. The steady-state ATPase activity in these reconstituted sarcomeres was analyzed by the oxidation of NADH to NAD+ to convert the ADP produced by active myosin S1 heads to ATP aided by pyruvate kinase and lactate dehydrogenase. The absorbance for NADH was measured at 340 nm in 25°C using a SpectraMax plate reader (Molecular Devices).

### Statistical analysis

A Shapiro-Wilk test was used to test for normality using the scipy python library (version 1.6.2) within JupyterLab Note-book (version 6.3.0) distributed through Anaconda (version 3). If *P >* 0.05, then we assumed a normal or parametric distribution. Statistical analyses for significance were performed using GraphPad Prism software (version 9.3) using either a 1- or 2-way analysis of variance (ANOVA), as applicable. For parametric distributions, ANOVA analyses used either Sidak's (comparing to a control) or Tukey’s (all pairwise) post hoc method for multiple pairwise comparisons. For nonparametric distributions, ANOVA analysis used a Kruskal-Wallis test post hoc method for multiple pairwise comparisons. A repeated measures ANOVA was used for within group 2-way ANOVA comparisons. A *P* value <0.05 was considered statistically significant. Data are represented as the group mean ± SEM.

## Results

### Small molecule selectivity

Both compounds activate the sarcomere to produce more contractile force. The rate of ATP consumed by the sarcomere during cross-bridge cycling is an indirect assessment of contractile force that can be measured by monitoring steady-state ATPase activity from myofibrils isolated from cardiac tissue. M (CK-138) and T (CK-030) selectivity were evaluated using a reconstituted hybrid sarcomere protein ATPase assay that was previously used to determine OM selectivity and, more recently, selectivity for CK-136, a small molecule that selectively alters troponin activity to increase cardiac muscle contraction.^48^ Steady-state myofibril ATPase activity was measured indirectly by monitoring the conversion of NADH to NAD+ by measuring the change in absorbance at 340 nm in presence of lactate dehydrogenase and pyruvate kinase.

The reconstituted sarcomere consisted of myosin S1 and thin filaments from either cardiac, fast, or slow skeletal muscle. Selectivity can be measured, because the molecules exhibit differential activity depending on the source of myosin and troponin. Here, we reconstituted an all-cardiac and an all-skeletal sarcomere as well as hybrid systems where myosin comes from cardiac but troponin comes from skeletal and vice versa. For M, we saw activity that tracked with the source of myosin. The reconstituted sarcomere with cardiac muscle myosin S1 had increased steady-state myosin ATPase activity in presence of 40 μmol/L M irrespective of thin filament origin (Supplemental Figure S1A). M is a structural analog of OM, and it is therefore not surprising that it exhibited a similar selectivity profile to OM.^54^ Similarly, T is a structural analog of CK-136 and was anticipated to exhibit a similar selectivity profile.^55^ For T, we saw activity that tracked with the source of troponin. T increased myosin ATPase activity in the reconstituted assays containing cardiac thin filaments as well as in reconstituted assays containing cardiac troponin irrespective of the source of myosin or tropomyosin (Supplemental Figures S1B and S1C). A modest degree of cross-reactivity between cardiac and slow skeletal thin filament was observed for T, as a modest increase in ATPase activity was observed for T using the slow skeletal thin filaments relative to fast skeletal thin filaments.

### M and T increased steady-state ATPase activity

**Figure 1:**
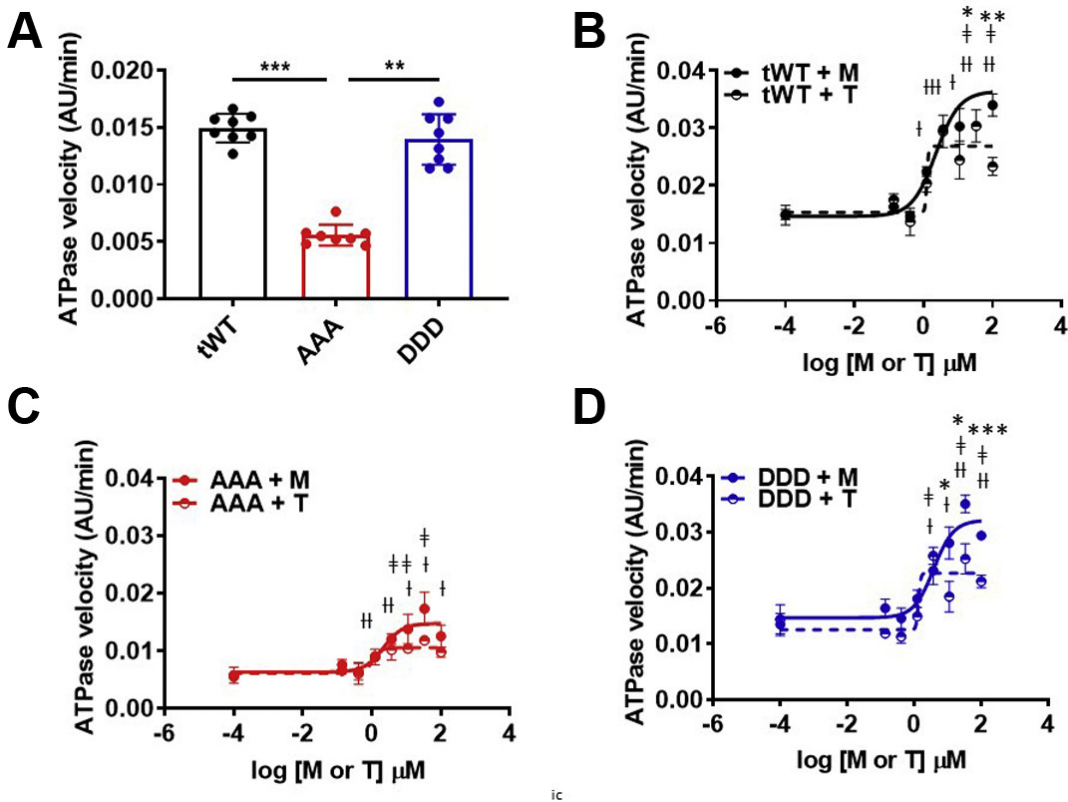
Myofibril Steady-State ATPase Activity **(A)** The steady-state ATPase activity in whole heart myofibrils isolated from tWt **(black)**, AAA **(red)**, and DDD **(blue)** transgenic mice. AAA myofibrils exhibit significantly lower steady-state ATPase activity when compared with tWt or DDD myofibrils (1-way analysis of variance, Kruskal-Wallis’ multiple comparison test [∗∗ *P <* 0.01, ∗∗∗ *P <* 0.001]). **(B to D)** Both the M **(filled circles)** and T **(half circles)** increased steady-state ATPase activity in myofibrils isolated from all 3 transgenic lines: tWt **(B),** AAA **(C),** and DDD **(D)** (2-way analysis of variance, Sidak’s multiple comparison test [†, ‡*P <* 0.05, ††,‡‡ *P <* 0.01, †††,‡‡‡ *P <* 0.001 for M and T, respectively]). M exhibited a modest but significant increase steady-state ATPase activity when compared with T (2-way analysis of variance, Sidak’s multiple comparison test [∗*P <* 0.05, ∗∗*P <* 0.01, ∗∗∗*P <* 0.001]). Data are represented as the group mean ± SEM. The curves were fitted to the equation of log(agonist) vs normalized response -variable slope 4 parameters variable slope model (GraphPad Prism 9.3). Four independent experiments were performed for myofibrils isolated from each transgenic line. AAA = transgenic mice expressing alanine at Serine 273, 282, and 302; DDD = transgenic mice expressing aspartate at Serine 273, 282, and 302; M = myosin-selective small molecule; T = troponin-selective small molecule; tWt = transgenic wild type.

Myofibrils isolated from tWt, AAA, and DDD transgenic mice were used to quantify steady-state ATPase activity to determine if alterations in cMyBP-C phosphorylation affected the activity of either M or T. Steady-state ATPase activity in a reaction containing 1.2 mmol/L calcium was significantly lower for AAA myofibrils compared with myofibrils isolated from tWt and DDD mice (Figure 1A). To assess the impact of M and T on basal steady-state myofibrillar ATPase activity, we next measured steady-state ATPase activity in the presence of increasing concentrations of either the M or T. Both compounds were able to significantly increase the steady-state ATPase activity across all 3 genotypes (Figures 1B to 1D, Table 1). M resulted in modest but statistically significant higher steady-state ATPase activity in tWt and DDD myofibrils at the highest concentrations relative to T in tWT and DDD myofibrils. At a maximal concentration of M, tWt myofibrils displayed a steady-state ATPase activity rate of 0.034 ± 9 × 10^−4^ AU/min, compared with a rate of 0.023 ± 7 × 10^−4^ AU/min in the presence of compound T (Figure 1B). For AAA isolated myofibrils, the steady-state ATPase activity rate was determined to be 0.013 ± 9 × 10^−4^ AU/min for M and 0.009 ± 4 × 10^−4^ AU/min for T. For DDD isolated myofibrils, the steady-state ATPase activity rate was 0.029 ± 3 × 10^−4^ AU/min for M and 0.021 ± 5 × 10^−4^ AU/min for T (Figures 1C and 1D, Table 1).

**Table 1:** Steady-State ATPase Activity Across Transgenic Cardiac Myofibrils

Genotype	Treatment	ATPase Activity (AU/min)
tWt	Control	0.015 ± 3 × 10^−3^
	M_max_	0.034 ± 9 × 10^−4^^a^
	T_max_	0.023 ± 7 × 10^−4^^b^^,^^c^
AAA	Control	0.005 ± 2 × 10^−3^
	M_max_	0.013 ± 9 × 10^−4^^b^
	T_max_	0.009 ± 4 × 10^−4^
DDD	Control	0.014 ± 6 × 10^−3^
	M_max_	0.029 ± 3 × 10^−4^^a^
	T_max_	0.021 ± 5 × 10^−4^^b^^,^^d^

Values are group mean ± SEM. Statistical analyses were performed in all groups using a 1-way analysis of variance, Tukey’s multiple comparison test with single pooled variance. Three myofibrillar ATPase experiments were performed independently.

AAA = transgenic mice expressing alanine at Serine 273, 282, and 302; DDD = transgenic mice expressing aspartate at Serine 273, 282, and 302; M = myosin-selective small molecule; T = troponin-selective small molecule; tWt = transgenic wild type.

a *P* ˂ 0.01 vs respective control.

b *P* ˂ 0.05 vs respective control.

c *P <* 0.01 between M and T treatment.

d *P <* 0.001 between M and T treatment.

### M and T increased cardiomyocyte cell shortening

To determine if M and T improved cell contractility, we measured cell shortening using cardiomyocytes isolated from tWt, AAA, or DDD transgenic lines with an IonOptix system. Preliminary work found that it required 3- to 4-fold less T relative to M to increase bovine myofibril turnover. For simplicity, we have referred to the concentrations for M and T as 1× and 3× throughout the paper, where 1× and 3× are 3.2 μmol/L and 9.6 μmol/L for M, whereas 1× and 3× are 1 μmol/L and 3 μmol/L for T.

**Figure 2:**
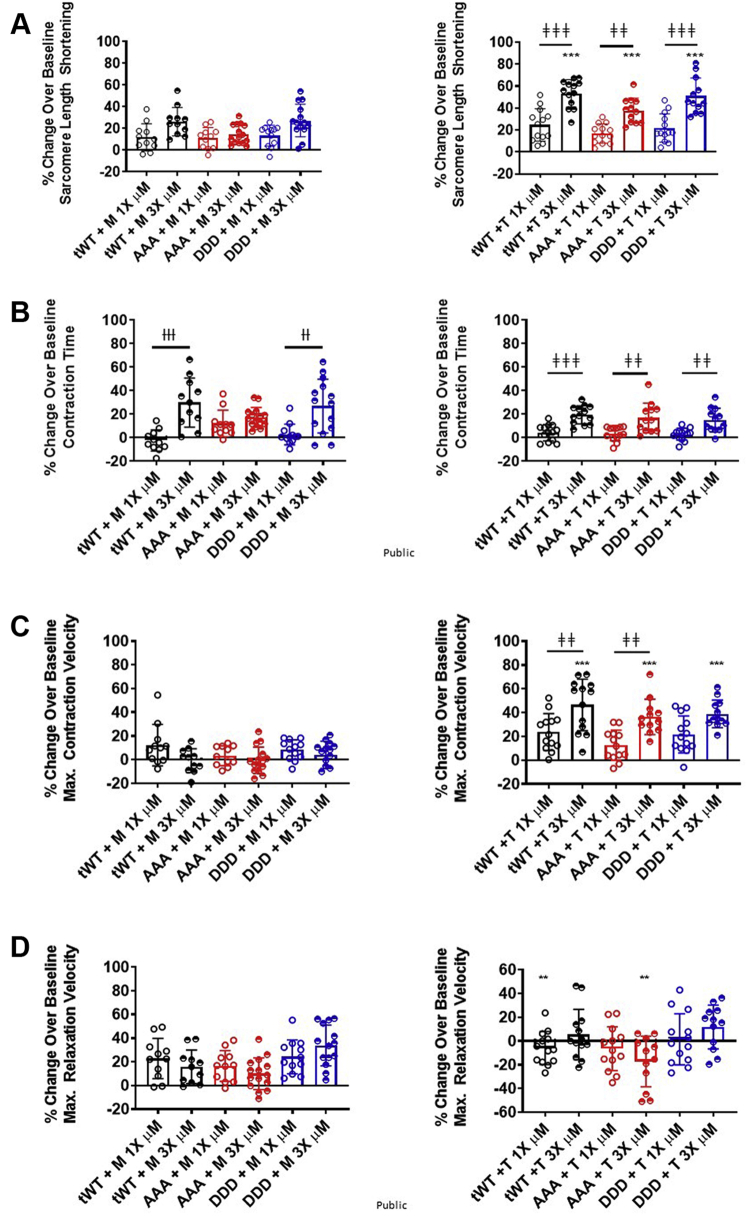
M and T Increased Cardiomyocyte Contraction Using Distinct Mechanisms Bar graph comparison of the % change over baseline in response to treatment for **(A)** cell shortening, **(B)** contraction time, **(C)** maximal contraction velocity and **(D)** maximal relaxation velocity in single cardiomyocytes isolated from Twt **(black)**, AAA **(red)**, and DDD **(blue)** mice, in presence of 1× **(open circles)** and 3× **(half-filled circles)** dose of M **(left)** and T **(right)**. Statistically significant values were calculated by performing a 1-way analysis of variance, Tukey’s multiple comparison test (†,‡,∗*P* < 0.05; ††, ‡‡, ∗∗*P* < 0.01; and †††, ‡‡‡, ∗∗∗*P* < 0.001 for 1× vs 3× M, 1× Vs 3× T, and M Vs T, respectively). Data represented as the group mean ± SEM. N = 11-15 cardiomyocytes per group. Cells were isolated from 2 to 4 animals for each group. Abbreviations as in Figure 1.

Isolated cardiomyocytes were pulsed through an electric field to induce contraction. The resting baseline sarcomere length was significantly shorter for cardiomyocytes isolated from AAA and DDD transgenic mice compared with tWt (Supplemental Figure S2). When we compared cells with a resting sarcomere length below the median to cells with a resting sarcomere length at or above the median for each genotype and treatment, we did not observe any obvious or consistent trend, suggesting that cells below the median sarcomere length were less responsive to treatment or influenced by genotype (Supplemental Figure S3). Consistent with the changes in steady-state ATPase activity, both compounds increased contraction relative to baseline contraction across all 3 genotypes. M increased percentage contraction, or cell shortening, in tWt (11.98% ± 3.76% and 26.27% ± 3.27%), AAA (10.99% ± 2.33% and 14.44% ± 2.30%), and DDD (13.36% ± 2.76% and 27.37% ± 4.00%) (Figure 2A). T increased percentage cell shortening in tWt (24.71% ± 4.08% and 53.42% ± 3.48%), AAA (16.87% ± 2.40% and 37.66% ± 3.30%), and DDD (21.96% ± 3.60% and 51.05% ± 4.70%) (Figure 2A). T at its 3× concentration (3 μmol/L) resulted in a significantly greater degree of cell shortening relative to 1× T or M at its 3× concentration (9.3 μmol/L) (Figure 2A).

The 3× dose of M led to a significantly larger relative increase in contraction time when compared with the 1× dose of M for tWt and DDD isolated cardiomyocytes, and T resulted in a significantly greater increase in contraction time at the 3× dose relative to the 1× dose of T for tWt, DDD and AAA isolated cardiomyocytes (Figure 2B). Additionally, the 3× dose of T led to a significantly greater increase in contraction velocity relative to the 1× dose of T in tWt and AAA isolated cardiomyocytes. This contrasts M, which elicited only a modest change in contraction velocity at both the 1× and 3× dose. The contraction velocity for the 3× dose of T was significantly greater relative to the 3× dose of M (Figure 2C). M resulted in a greater increase in the relaxation velocity at the 1× dose of T in tWt and the 3× dose of T in AAA isolated cardiomyocytes (Figure 2D). Collectively, these data indicate that both M and T increase cardiomyocyte contraction by increasing cell shortening irrespective of the proportion of cMyBP-C that is phosphorylated and suggest that they may do so through distinct mechanisms.

### M and T increased force generation in skinned cardiac papillary fibers

**Figure 3:**
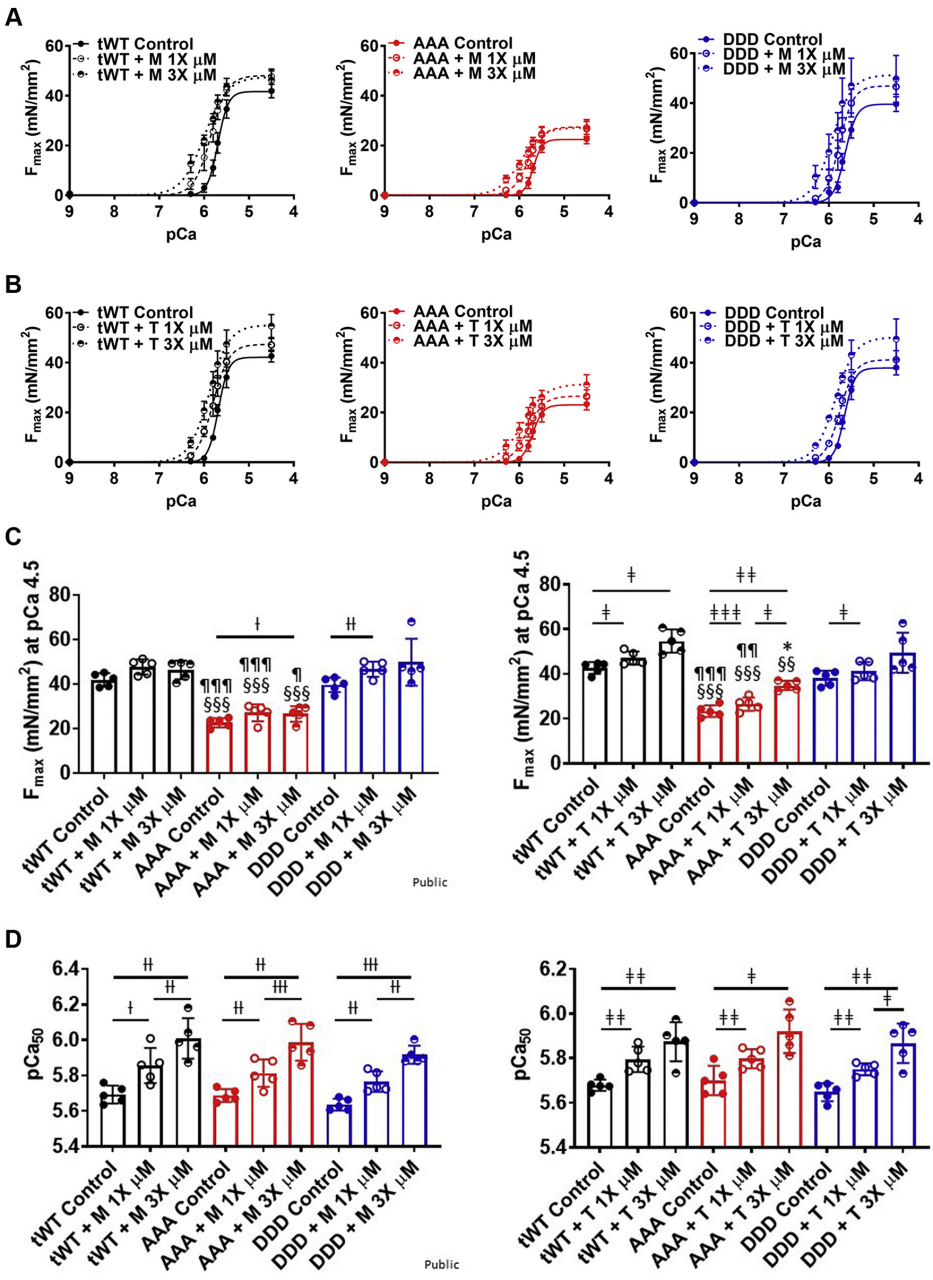
Force Generation in Skinned Cardiac Papillary Fibers **(A and B)** Maximal force vs the calcium concentration in presence of M or T produced in skinned papillary fibers isolated from tWt **(black),** AAA **(red),** and DDD **(blue)** at 1× dose **(empty circle, dashed line)** and 3× dose **(half-filled circle, dotted line). (C and D)** Bar graph comparison of maximal force produced at pCa 4.5 **(C)** and calcium sensitivity **(D)** in skinned cardiac papillary fibers isolated from tWt, AAA, and DDD transgenic mice in the presence of 1× and 3× dose of M and T. Statistically significant values were calculated by performing a 2-way analysis of variance, Tukey’s multiple comparison test (†, ‡, ∗*P <* 0.05; ††, ‡‡, ∗∗*P <* 0.01; and †††, ‡‡‡, ∗∗∗*P <* 0.001 for 1× vs 3× M, 1× vs 3× T, and M vs T, respectively; §*P <* 0.5, §§*P <* 0.01, §§§*P <* 0.001 untreated tWt vs AAA; ¶*P <* 0.05, ¶¶*P <* 0.01, ¶¶¶*P <* 0.001 untreated AAA vs DDD for the equivalent dose). n = 5 fibers per compound treatment per dosage made across fibers isolated from each transgenic line. Data represented as the group mean ± SEM. The equation used to fit the force curves was log(agonist) vs normalized response-variable slope (GraphPad Prism 9.3). Abbreviations as in Figure 1.

We next performed experiments measuring force production per cross-sectional area in skinned cardiac papillary fibers isolated from tWt, AAA, and DDD transgenic mice. The force produced in skinned papillary fibers was measured at 1× and 3× concentrations of M and T. Consistent with the myofibril ATPase activity assay results, maximal force (F_max_) produced by AAA papillary fibers (22.6 ± 1.9 and 23.4 ± 2.3 mN/mm^2^) at pCa 4.5 was significantly attenuated relative to tWt (41.9 ± 2.7 and 42.7 ± 2.4 mN/mm^2^) and DDD fibers (39.6 ± 3.0 and 38.1 ± 3.0 mN/mm^2^) (solid lines in Figures 3A to 3C, Table 2). The 1× and 3× dose of M did not result in a significant increase in F_max_ in tWt (47.7 ± 3.1 and 46.3 ± 3.8 mN/mm^2^), AAA (27.0 ± 3.4 and 26.6 ± 3.2 mN/mm^2^), and DDD (46.6 ± 3.0 and 49.8 ± 9.4 mN/mm^2^), except at the 3× dose in fibers isolated from AAA transgenic mice and the 1× dose in fibers isolated from DDD transgenic mice (Figure 3C, Table 2). A 1× and 3× dose of T resulted in a significant increase in the F_max_ for papillary fibers isolated from tWt (47.1 ± 2.6 and 54.7 ± 4.6 mN/mm^2^) and AAA (26.5 ± 2.6 and 31.2 ± 4.0 mN/mm^2^), whereas the 1× dose led to a significant increase in fibers isolated from DDD (41.2 ± 3.6 and 49.5 ± 8.0 mN/mm^2^) transgenic mice.

**Table 2:** Maximal Force, Calcium Sensitivity, Rate of Cross-Bridge Formation, and Drop in Maximal Force Across All Transgenic Mice Skinned Papillary Fibers With Compound M and T Treatment

Animal	Compound	Dosage	F_max_ (mN/mm^2^)	pCa_50_	k_tr_ (s^−1^)	Drop in F_max_ (%)
tWt	M	Control	41.9 ± 2.7^a^	5.69 ± 0.01	4.32 ± 0.18^b^	81.13 ± 6.3
		1×	47.7 ± 3.1^a^	5.86 ± 0.02^c^	4.31 ± 0.18^b^	68 ± 5.7^d^
		3×	46.3 ± 3.8^a^	6.02 ± 0.02^e^	4.05 ± 0.32^b^	30.94 ± 9.86^d^
	T	Control	42.7 ± 2.4^a^	5.68 ± 0.01	4.29 ± 0.13	83.13 ± 2.3
		1×	47.1 ± 2.6^a^^,^^f^	5.80 ± 0.01^g^	15.91 ± 2.68^h^^,^^l^	70.17 ± 6.66^f^
		3×	54.7 ± 4.6^g^^,^^i^	5.89 ± 0.01^f^	26.66 ± 3.78^h^^,^^l^	69.75 ± 7.12^g^
AAA	M	Control	22.6 ± 1.9^a^^,^^j^	5.69 ± 0.01	4.23 ± 0.15^j^	80.03 ± 6.2
		1×	27.0 ± 3.4^a^^,^^j^	5.82 ± 0.01^e^	4.42 ± 0.44^j^	68.5 ± 8.5^c^
		3×	26.6 ± 3.2^a^^,^^c^^,^^j^	6.00 ± 0.02^e^	4.35 ± 0.23^j^	30.05 ± 10.07^d^
	T	Control	23.4 ± 2.3^a^^,^^j^	5.70 ± 0.01	4.21 ± 0.19	83.03 ± 1.2
		1×	26.5 ± 2.6^a^^,^^k^^,^^l^	5.80 ± 0.01^f^	17.81 ± 3.59^h^	69.04 ± 6.20^f^
		3×	31.2 ± 4.0^g^^,^^i^	5.93 ± 0.02^f^	26.67 ± 3.09^h^^,^^l^	53.6 ± 10.11^f^
DDD	M	Control	39.6 ± 3.0^j^	5.64 ± 0.01	5.77 ± 0.23^b^^,^^j^	85.9 ± 8.9
		1×	46.6 ± 3.0^e^^,^^j^	5.77 ± 0.01^e^	5.88 ± 0.34^b^^,^^j^	73.84 ± 12.08
		3×	49.8 ± 9.4^j^	5.93 ± 0.01^d^	5.71 ± 0.44^b^^,^^j^	47.25 ± 22.4^c^
	T	Control	38.1 ± 3.0	5.65 ± 0.01	5.79 ± 0.18^b^^,^^j^	84.9 ± 3.4
		1×	41.2 ± 3.6 f^,^^k^	5.75 ± 0.01^f^	16.29 ± 2.46^h^^,^^l^	77.24 ± 8.97^f^
		3×	49.5 ± 8.0	5.88 ± 0.01^f^	26.59 ± 0.87^h^^,^^l^	70.38 ± 9.53^f^

Values are group mean ± SEM. Statistical analyses were performed in all groups using a 1-way analysis of variance, Tukey’s multiple comparison test. Data represents 5 fibers analyzed per animal per compound treatment isolated from 12- to 14-week-old mice. The pCa_50_ values were obtained for each individual fiber by fitting each of the fiber data to log(agonist) vs normalized response—variable slope model. The k_tr_ values were obtained for each fiber by fitting the curve to 1 phase association curve. The errors were obtained by comparing the fitted parameters for each fiber.

Abbreviations as in Table 1.

a *P <* 0.001 tWt vs AAA for the equivalent dose.

b *P <* 0.001 tWt vs DDD for the equivalent dose.

c *P <* 0.05 for control vs 1× and 3× M.

d *P <* 0.001 for control vs 1× and 3× T.

e *P <* 0.01 for control vs 1× and 3× M.

f *P <* 0.05 for control vs 1× and 3× T.

g *P <* 0.01 for control vs 1× and 3× T.

h *P <* 0.001 for M vs T for the equivalent dose.

i *P <* 0.01 tWt vs AAA for the equivalent dose.

j *P <* 0.001 and

k *P <* 0.01 AAA vs DDD for the equivalent dose.

l *P <* 0.001 for control vs 1× and 3× M.

We also measured the effect of M and T on the calcium sensitivity (pCa_50_) of the fibers. For papillary fibers isolated from tWt (5.69 ± 0.01 and 5.68 ± 0.01), AAA (5.69 ± 0.01 and 5.7 ± 0.01), and DDD (5.64 ± 0.01 and 5.65 ± 0.01) transgenic lines, there was no significant difference found between the pCa_50_ in untreated fibers (Figure 3D, Table 2). M and T at 1× and 3× doses were able to significantly increase the pCa_50_ of papillary fibers across all 3 genotypes. 1× and 3× of M increased the pCa_50_ in tWt (5.86 ± 0.02 and 6.02 ± 0.02), AAA (5.82 ± 0.01 and 6.00 ± 0.02), and DDD (5.77 ± 0.01 and 5.93 ± 0.01), respectively. 1× and 3× of T also increased the pCa_50_ in tWt (5.80 ± 0.01 and 5.89 ± 0.01), AAA (5.80 ± 0.01 and 5.93 ± 0.02), and DDD (5.75 ± 0.01 and 5.88 ± 0.01), respectively. The increase in calcium sensitivity upon compound treatment indicates an improvement in force-producing capability for skinned fiber at low calcium concentrations.

### T exhibited a faster tension redevelopment rate

**Figure 4:**
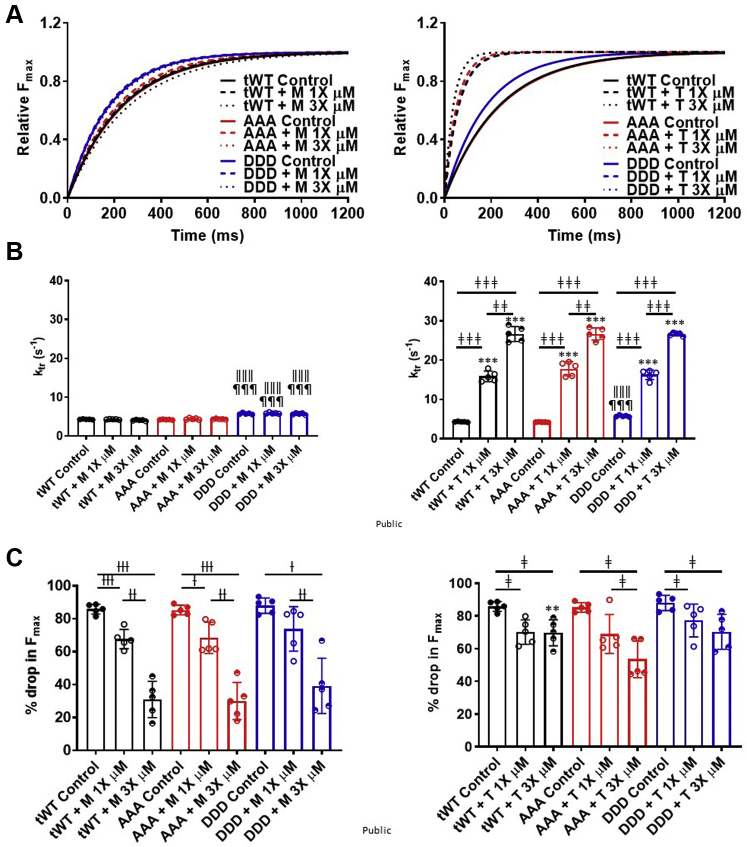
T Increased the Tension Redevelopment Rate (K_tr_) **(A)** One-phase association fit curves for rate of force redevelopment in skinned papillary fibers isolated from transgenic tWt **(black),** AAA **(red),** and DDD **(blue)** at 1× **(dashed)** and 3× **(dotted)** concentrations of M **(left)** and T **(right). (B)** Bar graph comparison for *k*_*tr*_ values across tWt, AAA, and DDD muscle fibers in presence of M **(left)** and T **(right)** at 1× **(open circles)** and 3× **(half-filled circles)** concentrations. **(C)** Bar graph comparison of the drop in maximal force across tWt, AAA, and DDD transgenic muscle fibers in presence of M **(left graph)** and T **(right graph)** at 1× **(open circles)** and 3× **(half-filled circles).** Statistically significant values were calculated by performing a 2-way analysis of variance, Tukey’s multiple comparison test (†, ‡, ∗*P <* 0.05; ††, ‡‡, ∗∗*P <* 0.01; and †††, ‡‡‡, ∗∗∗*P <* 0.001 for 1× vs 3× M, 1× vs 3× T, and M vs T, respectively; ǁ*P <* 0.5, ǁǁ*P <* 0.01, ǁǁǁ*P <* 0.001 untreated tWt vs DDD; ¶*P <* 0.05, ¶¶*P <* 0.01, ¶¶¶*P <* 0.001 untreated AAA vs DDD for the equivalent dose). n = 5 fibers per compound treatment in fibers isolated from each transgenic line. Data represented as the group mean ± SEM. The curves were fitted to 1 phase association model (GraphPad Prism 9.3).

The effect of M and T on the force, or tension, redevelopment rate (*k*_tr_) was also measured at a submaximal calcium concentration, pCa 5.7. The *k*_tr_ for DDD displayed a significantly higher *k*_tr_ (5.77 ± 0.23 and 5.79 ± 0.18 s^−1^) relative to AAA (4.23 ± 0.15 and 4.21 ± 0.19 s^−1^) or tWt (4.32 ± 0.18 and 4.29 ± 0.13 s^−1^) (Figures 4A and 4B, Table 2). M at 1× and 3× concentrations did not significantly alter the *k*_tr_ across all genotypes (tWt [4.31 ± 0.18 and 4.05 ± 0.32 s^−1^], AAA [4.42 ± 0.44 and 4.35 ± 0.23 s^−1^], and DDD [5.88 ± 0.34 and 5.71 ± 0.44 s^−1^]) (Figures 4A and 4B, Table 2). In contrast, T exhibited significantly faster rates of tension redevelopment rates (*k*_tr_) across all genotypes (tWt [15.91 ± 2.68 and 26.66 ± 3.78 s^−1^], AAA [17.81 ± 3.59 and 26.67 ± 3.09 s^−1^], and DDD [16.29 ± 2.46 and 26.59 ± 0.87 s^−1^]) (Figures 4A and 4B, Table 2).

### M and T hindered the release of the myosin head from the actin thin filament

We also measured the percentage drop in force in fibers during the 20% slack portion of the force redevelopment protocol. The percentage drop in force is indicative of the degree to which the myosin heads release the actin thin filament during slack. A comparable percentage drop in force was observed for untreated fibers isolated from all 3 transgenic lines (tWt [81.13% ± 6.3% and 83.13% ± 2.3%], AAA [80.03% ± 6.2% and 83.03% ± 1.2%], and DDD [85.9% ± 8.9% and 84.9% ± 3.4%]). Representative traces from untreated and from M- and T-treated fibers illustrate the prolonged attachment of the myosin heads to actin, which impeded detachment leading to a significant reduction in the % drop in F_max_ when compared with untreated (Supplemental Figures S4A and S4B). The 1× and 3× concentration of T significantly reduced the drop in force (tWt [70.17% ± 6.66% and 69.75% ± 7.12%], AAA [69.04% ± 6.20% and 53.6% ± 10.11%], and DDD [77.24% ± 8.97% and 70.38% ± 9.53%]). M also resulted in a lower drop in force, or myosin head release at 1× and 3× (tWt [68.0% ± 5.7% and 30.94 ± 9.86%], AAA (68.5% ± 8.5% and 30.05% ± 10.07%], and (73.84% ± 12.08% and 47.25% ± 22.4%), respectively) (Figure 4C, Table 2). This suggests that the acto-myosin cross-bridges do not break as easily, and a proportion are bound for a prolonged period. Another observation that supports the impediment for detachment of myosin from actin thin filaments was made during the washout step of calcium in skinned papillary muscle fibers. The force generated in muscle fibers at maximal pCa 4.5 rapidly decays when exchanged with pCa 9.0 (Supplemental Figure S4C, black). Here, treatment with T slowed force decay relative to untreated fibers, and M treatment slowed force decay further when compared with control and T treatment (Supplemental Figure S4C, red and blue).

### M slowed mANT-ATP exchange

We next wanted to compare ATP turnover rates, because it has been reported that OM prolongs the duration of the strongly bound state.^56^^,^^57^ To assess the rate of ATP turnover, we monitored mANT-ATP fluorescence decay. In these experiments, in the presence of M or T, the permeabilized LV muscle fiber was conformed to a rigor state with rigor buffer in the absence of ATP. mANT-ATP was added followed by ATP to allow for mANT-ATP binding to the sarcomere and subsequent exchange with ATP. M and T were included at each stage of the experiment. The exchange of mANT-ATP with ATP exhibited a 2-phase exponential decay in fluorescence, yielding 2 exchange states. M and T did not cause photobleaching of mANT-ATP (Supplemental Figure S5A) and did not significantly affect the mANT-ATP retention by permeabilized LV muscle fibers (Supplemental Figure S5B).

**Figure 5:**
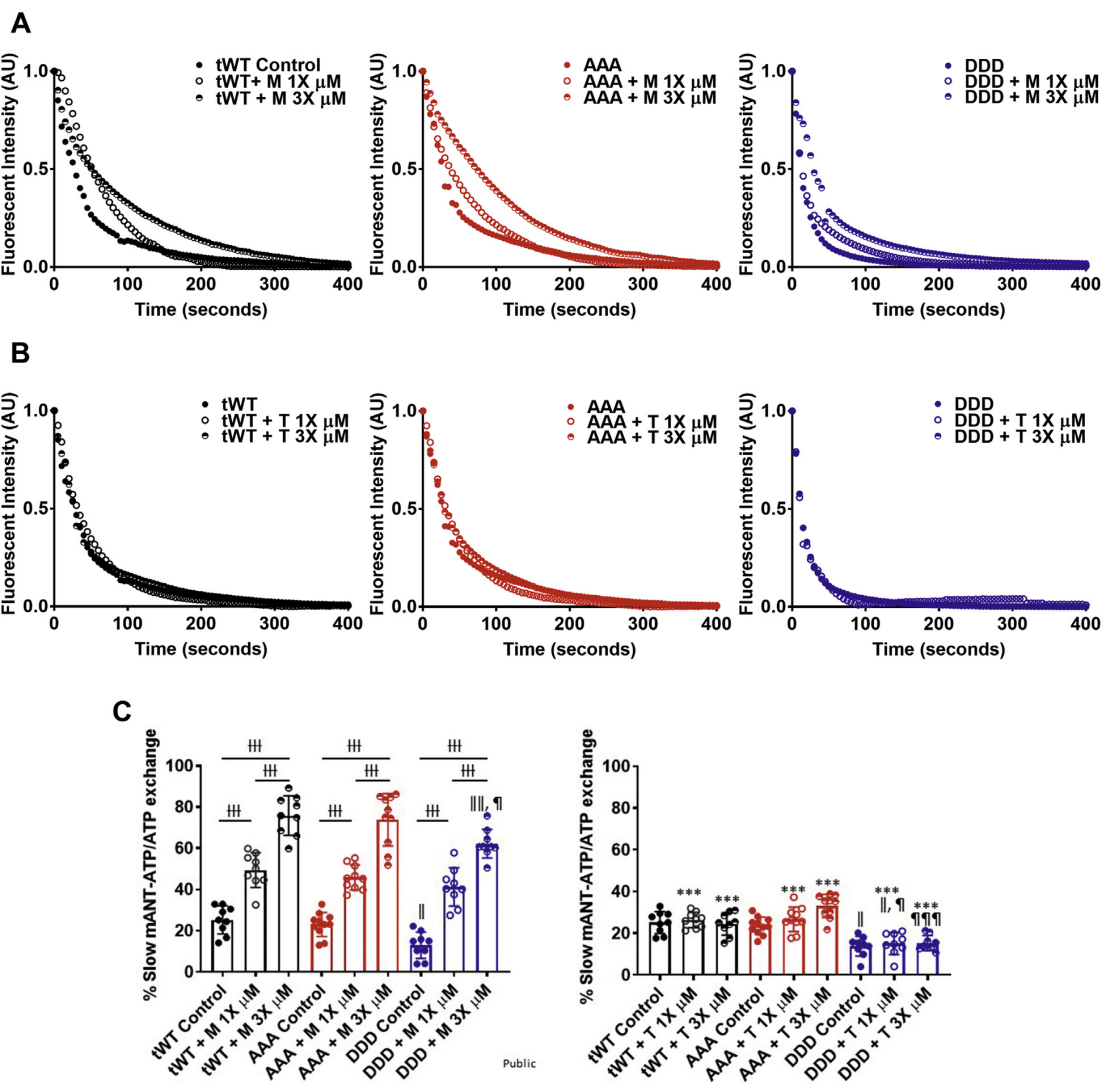
M Decreased the Mant-ATP Exchange Rate in Skinned Left Ventricle Muscle Fibers Raw traces of fluorescent decay for Mant-ATP exchange with ATP in Twt **(black)**, AAA **(red)**, and DDD **(blue)** in the presence of **(A)** M and **(B)** T at 1× **(open circles)** and 3× **(half-filled circles)** concentrations. **(C)** Bar graphs comparing the slow phase fluorescent decay across Twt, AAA, and DDD muscle fibers in the presence of M **(Left)** and T **(Right)** at 1× **(open circles)** and 3× **(half-filled circles)** concentrations. Statistically significant values were calculated by performing a 1-way analysis of variance, Tukey’s multiple comparison test (†, ‡ *P* < 0.05; ††, ‡‡ *P* < 0.01; and †††, ‡‡‡ *P* < 0.001 for 1× vs 3× M And 1× vs 3× T, respectively; ǁ*P* < 0.5, ǁǁ *P* < 0.01, ǁǁǁ *P* < 0.001 untreated Twt vs DDD; ¶ *P* < 0.05, ¶¶ *P* < 0.01, ¶¶¶ *P* < 0.001 untreated AAA vs DDD for the equivalent dose). N = 9-11 fibers per compound treatment in fibers isolated from each transgenic line. Data represented as the group mean ± SEM. The curves were fitted to custom 2-phase exponential decay model.

The slow phase for mANT-ATP turnover, or exchange, was comparable for fibers isolated from tWt (24.6% ± 0.8% and 23.8% ± 0.7%) and AAA (27.6% ± 1.3% and 25.6% ± 0.6%) mice but was significantly lower in fibers isolated from DDD mice (12.98% ± 0.4% and 14.1% ± 0.4%)^37^ (Supplemental Figures S5C and S5D). M at both the 1× and 3× concentrations contributed to a significant increase in the proportion of slow mANT-ATP exchange (tWt [51.7 ± 1.2% and 69.18 ± 2.4%], AAA [51.4% ± 2.4% and 55.08% ± 3.2%], and DDD [41.21% ± 2.9% and 62.25% ± 2.2%]) (Figures 5A to 5C, Table 3). In contrast, T did not significantly increase the mANT-ATP slow phase exchange rate (Figures 5B and 5C, Table 3). Changes to the proportion of muscle fibers with fast mANT-ATP exchange were inversely related to the observed changes in the phase for slow mANT-ATP exchange (Supplemental Figure S5A, Supplemental Table S1). The change in lifetimes for each of the fast and the slow phases were not significant upon M and T treatment (Supplemental Figures S6B and S6C, Supplemental Table S1).

**Table 3:** Percentage of Myosin Population for Slow Turnover of mANT-ATP With ATP

Animal	Compound	Dosage	% for Slow mANT-ATP Exchange
tWt	M	Control	24.6 ± 0.8^a^
		1×	51.7 ± 1.2^b^
		3×	69.18 ± 2.4^b^^,^^c^
	T	Control	23.8 ± 0.7^a^
		1×	36.05 ± 5.6^a^^,^^d^
		3×	25.11 ± 0.89^d^
AAA	M	Control	27.59 ± 1.3
		1×	51.4 ± 2.4^b^
		3×	55.08 ± 3.2^b^^,^^e^
	T	Control	25.59 ± 0.6^e^
		1×	28.74 ± 1.1^d^
		3×	51.84 ± 4.6^d^^,^^f^
DDD	M	Control	12.98 ± 0.4^a^
		1×	41.21 ± 2.9^b^
		3×	62.25 ± 2.2^b^^,^^c^^,^^e^
	T	Control	14.1 ± 0.4^a^
		1×	21.38 ± 1.6^a^^,^^d^^,^^e^
		3×	36.34 ± 6.9^d^^,^^f^

Values are group mean ± SEM. Statistical analyses were performed in all groups using a one-way analysis of variance (ANOVA), Tukey’s multiple comparison test with single pooled variance. Five left ventricle fibers were analyzed per animal per compound concentration.

Abbreviations as in Table 1.

a *P <* 0.50 tWt vs DDD for the equivalent dose.

b *P <* 0.001 for control vs 1× T.

c *P <* 0.01 tWt vs DDD for the equivalent dose.

d *P <* 0.001 for M vs T for the equivalent dose.

e *P <* 0.05 and

f *P <* 0.001 AAA vs DDD for the equivalent dose.

## Discussion

This study evaluated 2 new small molecules that target 2 different sarcomere proteins to increase contractility. M, a myosin targeting small molecule surrogate of OM, modulates the activity of myosin heads, while T, a surrogate of CK-136, targets the troponin complex. Both compounds result in a leftward shift of the force-calcium curve, indicating an ability of the muscle to produce more force for a given calcium concentration to augment cardiac muscle contraction efficiency.

cMyBP-C phosphorylation is a common post-translational modification associated with HF where reduced phosphorylation impairs contractility by decreasing the number of myosin heads that are actively engaged in cross-bridge cycling. Given the fact that cMyBP-C is more than 50% dephosphorylated in failing hearts regardless of disease etiology,^58^^,^^59^ it is of great importance to determine the ability to modulate the activity of these sarcomere proteins irrespective of the phosphorylation state.

cMyBP-C phosphorylation status did not alter the effectiveness of either M or T. Nevertheless, maximal force remains attenuated in the AAA transgenic line, because reducing the level of phosphorylated cMyBP-C reduced the population of myosin heads actively undergoing cross-bridge cycling. Our data therefore indicate that both M and T increase force generation by modulating cross-bridge cycling for active myosin heads. Our work is largely consistent with recently published work that evaluated the effect of OM on force generation using myocardial tissue isolated from wild-type and phosphorylation-deficient cMyBP-C mice.^60^ Our work extends this work with the inclusion of measures of cross-bridge cycling and the evaluation of a new small molecule class that alter troponin activity to increase cardiac muscle contraction.

This study revealed key differences related to the mechanism used by M and T to increase contractile force generation. The force mechanic experiments showed that T contributed to a much faster tension redevelopment rate (*k*_tr_) and suggest that, at concentrations near their respective in vitro EC_50_s, T led to a greater rate of ATP exchange. The dose dependent decline in the percent drop in F_max_ with M at these concentrations upon slack provides additional evidence, as the drop in F_max_ is indicative of myosin head - thin filament release and suggests that M impeded this action.

A delay in relaxation because of M manifests as a prolongation of systole and, at high doses, a curtailment of diastole in vivo.^61^^,^^62^ At therapeutically relevant doses, this extra squeeze by the heart through a prolongation of contraction will improve cardiac performance.^13^^,^^14^^,^^17^ It seems reasonable to conclude that the prolongation of systole at the expense of diastole would translate to a narrowing of the therapeutic window for a molecule that alters myosin activity relative to a molecule that alters troponin activity.

Previous data have shown that OM reduced the population of myosin in the super relaxed, or SRX, confirmation.^12^ This was determined by measuring the orientation of fluorescently labelled myosin RLC relative to the thick-filament axis in the absence and presence of OM. mANT-ATP exchange has also been used previously to assess the proportion of myosin heads in SRX, as a higher proportion of myosin heads in SRX will result in a slower ATP exchange rate.^46^ Our data revealed that OM increased the slow exchange phase and decreased the fast exchange phase suggesting an increase in the proportion of myosin heads in SRX and seemingly contradicting the myosin head orientation studies performed with OM. However, it is important to note that our studies were conducted using fibers in the presence of M or T before and during mANT-ATP exchange, and we interpret the observed decrease in mANT-ATP exchange in fibers incubated with M within the context of previous work that found that OM slowed the rate of phosphate release.^12^^,^^60^^,^^63^ Within this context, we propose that M has not led to a higher proportion of myosin heads in SRX but has slowed the rate of mANT-ATP exchange in a manner that is similar to its previously reported ability to slow the rate of phosphate release. In this context, we speculate that M has not altered the myosin head confirmation but has altered the affinity of mANT-ATP for myosin thereby impeding the exchange rate. Alternatively, it remains possible that CK-138 alters the proportion of myosin heads in SRX in a manner that is fundamentally different relative to OM. However, it is worth noting that CK-138 and OM are structurally very similar to one another. The only difference is at a single position where a hydrogen atom is replaced with fluorine.^54^ We feel that this substitution is unlikely to lead to such a large change in activity between OM and CK-138, given that a comparison of the 2 molecules, to date, suggest that they exhibit a similar activity profile with OM being more potent.

Our data indicate that both molecules sensitize the cardiac muscle to calcium resulting in an increase in the force produced by cardiac muscle to improve ejection fraction. These data support their use for patients with HF with reduced ejection fraction. Another potential use for the molecules would be for the treatment of dilated cardiomyopathy caused by allelic variants in calcium-handling proteins like phospholamban^64^ or cardiac arrythmia caused by an abnormal response to calcium.^63^ These molecules sensitize the cardiac muscle to calcium, thus allowing more work to be performed per calcium ion available.

### Study limitations

First, the transgenic lines used in these experiments overexpressed the phospho-mimetic (DDD), the phosphorylation deficient (AAA), and wild-type cMyBP-C (tWt). Although these lines have been well characterized,^26^^,^^36^ a preferred method for introducing these allelic variants would be to directly alter the cMyBP-C gene. Ex vivo pressure-volume loop measurements could have helped further elucidate the diastolic and systolic pressure changes in presence of M and T. Additionally, our results were obtained under conditions of steady Ca^2+^ activation. This stands in contrast to changes in Ca^2+^ concentration, or flux, that occur during the normal cardiac cycle.

Furthermore, it is worth noting that cMyBP-C is itself an activator of muscle. Recent studies have shown that the N-terminal fragment of cMyBP-C increases force in permeabilized cardiomyocytes^32^ and can improve cardiac performance in cMyBP-C KO mice.^61^ Neither small molecule targets cMyBP-C directly. Therefore, the small molecules are not anticipated to evoke full activation of the thick filament.

Additionally, translatability is always a concern. The studies were performed primarily on mice, where MYH6 is the dominant form of cardiac myosin, whereas humans express the MYH7 variant. M is a surrogate of OM, and OM has been shown to be *MYH7* selective.^65^ It, therefore, seems reasonable to infer that M may be exhibit more robust activity in humans. Finally, it remains to be seen how a molecule that alters troponin activity to increase cardiac contraction will perform in the clinic. Our data collectively suggest that targeting troponin may result in a differentiating therapeutic profile.

Perspectives **COMPETENCY IN MEDICAL KNOWLEDGE**: HF is a grievous condition with poor prognosis. Current treatments that reduce HF-related death and hospitalizations are focused on antagonizing the neurohormonal and hemodynamic stress axes. Directly targeting sarcomere proteins represents a potentially new therapeutic class for HF management that directly target the basic functional unit of cardiac muscle to increase contraction efficiency.

**TRANSLATIONAL OUTLOOK**: This work provides evidence that selectively activating different proteins within the sarcomere can increase muscle contraction by different means. We believe this could result in differentiating therapeutic profiles for this future class of medicines.

## Funding Support and Author Disclosures

Dr Sadayappan has received support from National Institutes of Health grants R01 AR078001, R01 HL130356, R01 HL105826, R38 HL155775, and R01 HL143490; and provides consulting and collaborative research studies to the Leducq Foundation (CURE-PLAN), Red Saree Inc, Greater Cincinnati Tamil Sangam, Novo Nordisk, Pfizer, AavantiBio, AstraZeneca, MyoKardia, Merck, and Amgen, but such work is unrelated to the content of this work. All other authors were employed by Amgen at the time the experiments were conducted.
