# Supplementary material for: Distinct Mechanisms for Increased Cardiac Contraction Through Selective Alteration of Either Myosin or Troponin Activity
Source: JACC Basic Transl Sci. 2022 Sep 7;7(10):1021–37. doi: 10.1016/j.jacbts.2022.04.013 (PMC9626889; doi:10.1016/j.jacbts.2022.04.013)
Supplement: Supplemental Figures 1–6 Table 1 [file mmc1.pdf]

Supplemental Appendix

Distinct mechanisms for increased cardiac contraction through selective alteration of either myosin or troponin activity

Rohit R. Singh *[a,b], Rebecca Slater *[a], Jinghong Wang [a], Chen Wang [a], Qi Guo [a], Alykhan S. Motani [a], James Hartman [c], Sakthivel Sadayappan [b] and Brandon Ason [a].

a) Department of Cardiometabolic Disorders, Amgen, South San Francisco, California – 94080, USA.
b) Department of Internal Medicine, University of Cincinnati, Cincinnati, Ohio - 45267, USA.
c) Discovery Biology, Cytokinetics, South San Francisco, California – 94080, USA.

To whom correspondence should be addressed: Rohit R. Singh, PhD. E-mail: rsingh14@amgen.com and Brandon Ason, PhD. E-mail: bason@amgen.com

Supplemental figures with accompanying figure legends – pages 2-8

Supplemental Figure 1: page 2

Supplemental Figure 2: page 3

Supplemental Figure 3: page 4

Supplemental Figure 4: page 5

Supplemental Figure 5: page 6

Supplemental Figure 6: pages 7-8

Supplemental tables – page 9

Supplemental Table 1: page 9

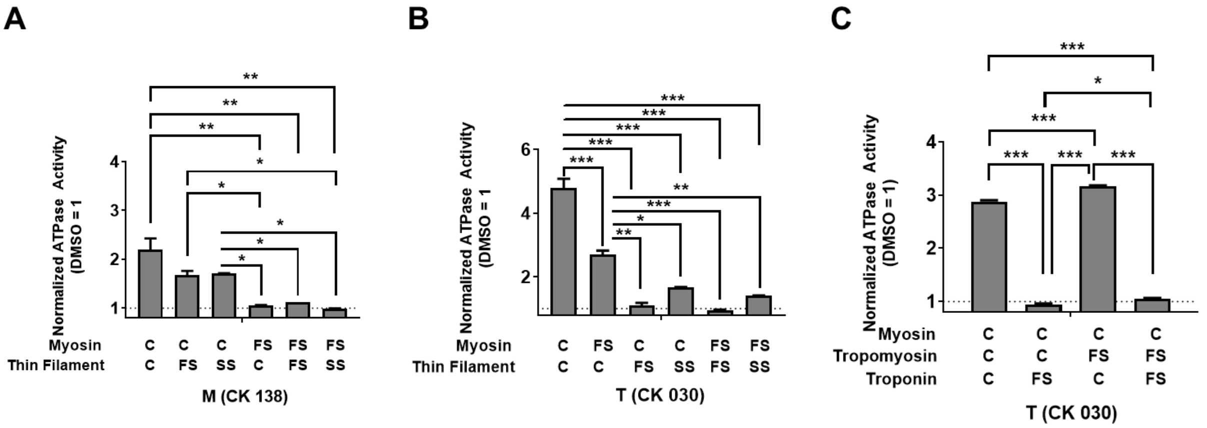

Supplemental Figure S1. M and T selectivity. Bar graphs for normalized ATPase activity in reconstituted sarcomeres with varying sources for myosin and thin filaments. The different striated muscle sources utilized were C = cardiac; FS = fast skeletal; SS = slow skeletal. (**A**) M (CK-138) (40 μM) selectively activates cardiac myosin irrespective of thin filament source. (**B**) T (CK-030) (40 μM) selectively activates cardiac thin filaments irrespective of myosin source. Assays containing slow skeletal thin filaments are activated to a lesser extent. (**C**) T (CK-030) (40 μM) selectively activates cardiac troponin. Statistically significant values were calculated by performing a one-way analysis of variance (ANOVA), Tukey's multiple comparison test, where * $p<0.05$, ** $p<0.01$ and *** $p<0.001$. Data represented as the group mean ± the standard error mean (SEM), n= 2-3 replicate reactions, where values above 1 signify the effect of sarcomere contractility over myosin ATPase activity.

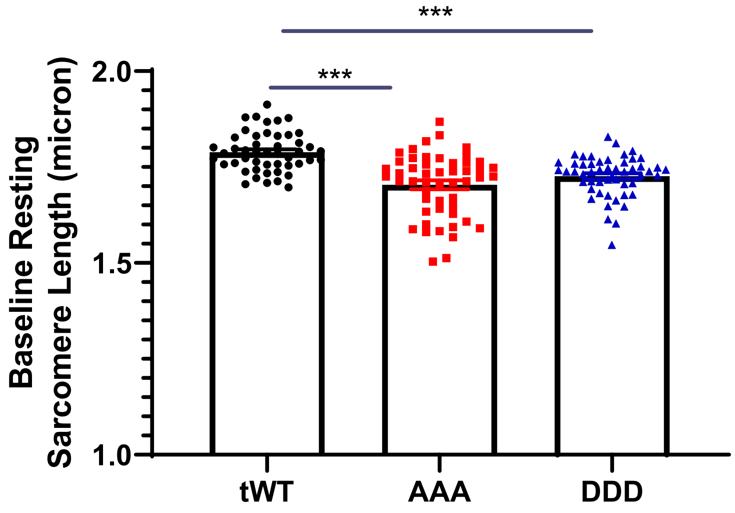

Supplemental Figure S2. cMyBPC-3 transgenic mice displayed shorter sarcomere length at baseline. Bar graph comparison for sarcomere length in single cardiomyocytes isolated from tWT (black circles), AAA (red squares) and DDD (blue triangles) transgenic mouse hearts. Statistically significant values were calculated by performing a one-way analysis of variance (ANOVA), Tukey's multiple comparison test, where * $p<0.05$, ** $p<0.01$ and *** $p<0.001$. N=48, 51, 51 for the number of tWT, AAA and DDD cardiomyocytes evaluated from each transgenic line, respectively. Cells were isolated from two to four animals for each group. Data represented as the group mean ± the standard error mean (SEM).

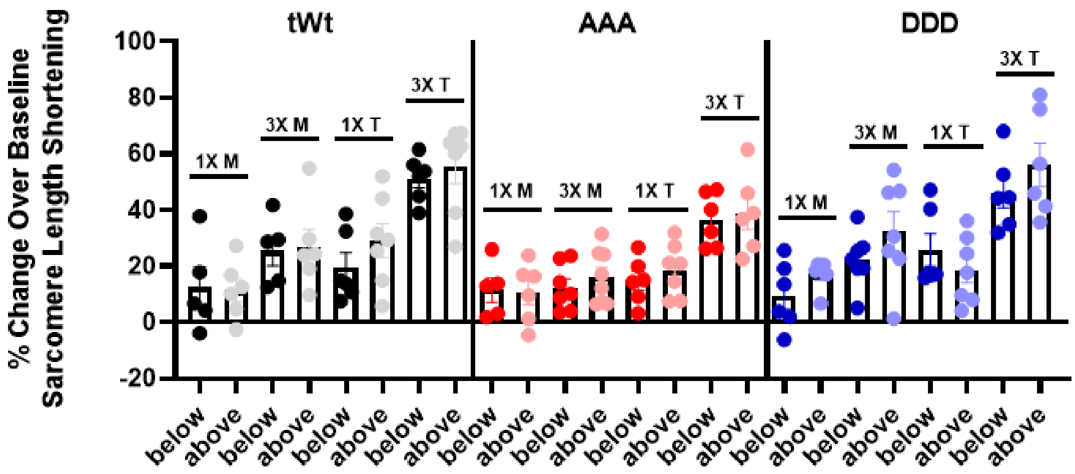

Supplemental Figure S3. Comparison of the response to M and T split by baseline sarcomere length. Bar graph comparison for cardiomyocytes isolated from tWT (black and grey), AAA (red and light red) and DDD (blue and light blue) mouse hearts. Cells with baseline sarcomere length below the median (below) are black, red and blue filled circles for tWt, AAA and DDD transgenic lines, respectively. Cells with a baseline sarcomere length at or above the median (above) are grey, light red and light blue filled circles for tWt, AAA and DDD transgenic lines, respectively. Statistically significant values were calculated by performing a one-way analysis of variance (ANOVA), Tukey's multiple comparison test, where * $p<0.05$, ** $p<0.01$ and *** $p<0.001$. Data represented as the group mean ± the standard error mean (SEM).

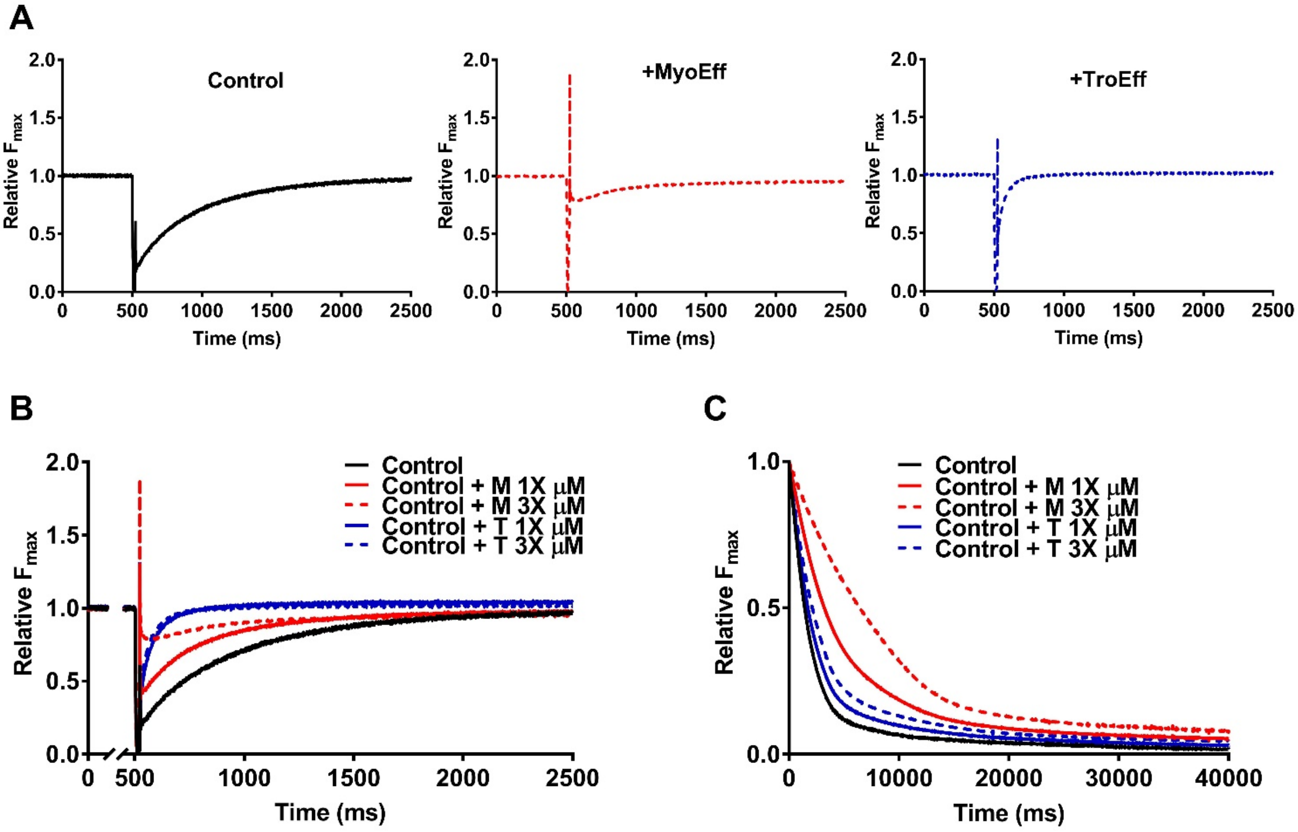

Supplemental Figure S4. **M and T impeded the myosin heads detachment from actin thin filaments.** (**A**) Representative figure for comparison of raw traces for the force produced by permeabilized papillary fibers during the force redevelopment protocol. At the 500 ms mark, the slack is introduced for 20 ms, which allows the force to drop in control (black), M treated (red dashed) and T treated (blue dashed) fibers. All figures are created with 3X treatment to showcase the effect on the force decay during rate of force redevelopment and calcium washout in permeabilized papillary fibers. (**B**) Representative figure for comparison of force drops compared to control (black) with fibers treated with M 1X (red) and 3X (red dashed) as well as fibers treated with T 1X (blue) and 3X (blue dashed) concentration. M at both concentrations impedes the detachment of myosin from actin thin filaments. **(C)** Representative figure for comparison of force decay when permeabilized fibers are recycled back to pCa 9.0 after cycling through increasing calcium concentration to washout calcium. M at both concentrations (red, red dashed) doesn't allow force to decay at the same rate as that of control (black) and T (blue and blue dashed) treatment.

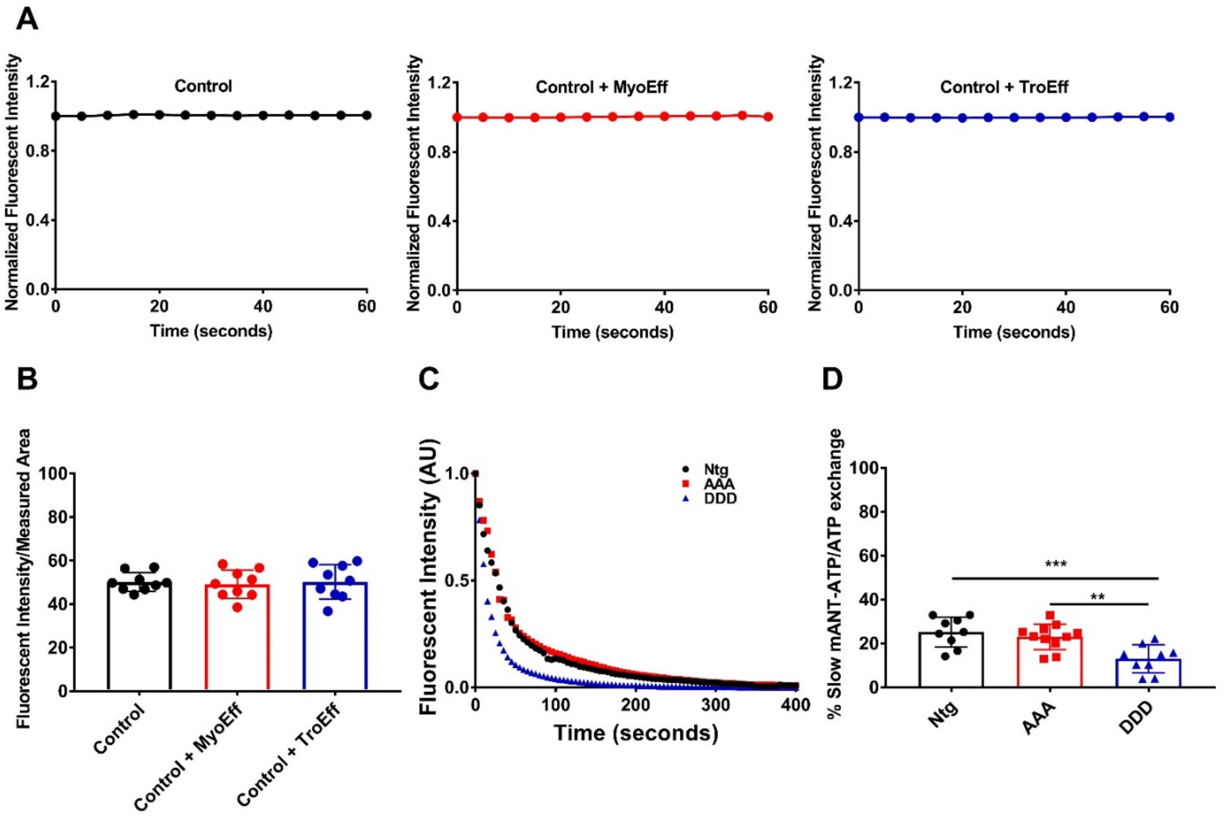

Supplemental Figure S5. M and T did not impact mANT-ATP retention by permeabilized LV muscle fibers with no measurable photobleaching (**A**) Representative figure for fluorescence observed for 60 seconds in permeabilized LV muscle fibers after mANT-ATP incubation to detect photobleaching. No photobleaching of mANT-ATP was observed in control (black left), M (red middle) and T (blue right) treatment. (**B**) Representative figure for initial fluorescence observed in permeabilized LV muscle fibers to detect any change in fluorescence or retention of mANT-ATP by M or T treatment. Compared to control (black), no significant change was observed for mANT-ATP fluorescence in muscle fibers with M (red) and T (blue) treatment. Both panels (**A**) and (**B**) panels were created with 3X treatment to showcase its effect on the mANT-ATP fluorescence. (**C**) Raw traces of fluorescent decay for exchange of mANT-ATP with ATP in tWt (black circles), AAA (red squares) and DDD (blue triangles) skinned left ventricle muscle fibers. (**D**) Bar graph comparison for population of myosin in slow phase fluorescent decay, across tWt, AAA and DDD muscle fibers. Statistically significant values were calculated by performing a one-way analysis of variance (ANOVA), Tukey's multiple comparison test, where * $p<0.05$, ** $p<0.01$ and *** $p<0.001$. N=9-11 fibers isolated from each transgenic line. Data represented as the group mean ± the standard error mean (SEM). The curves were fitted to two phase exponential decay model.

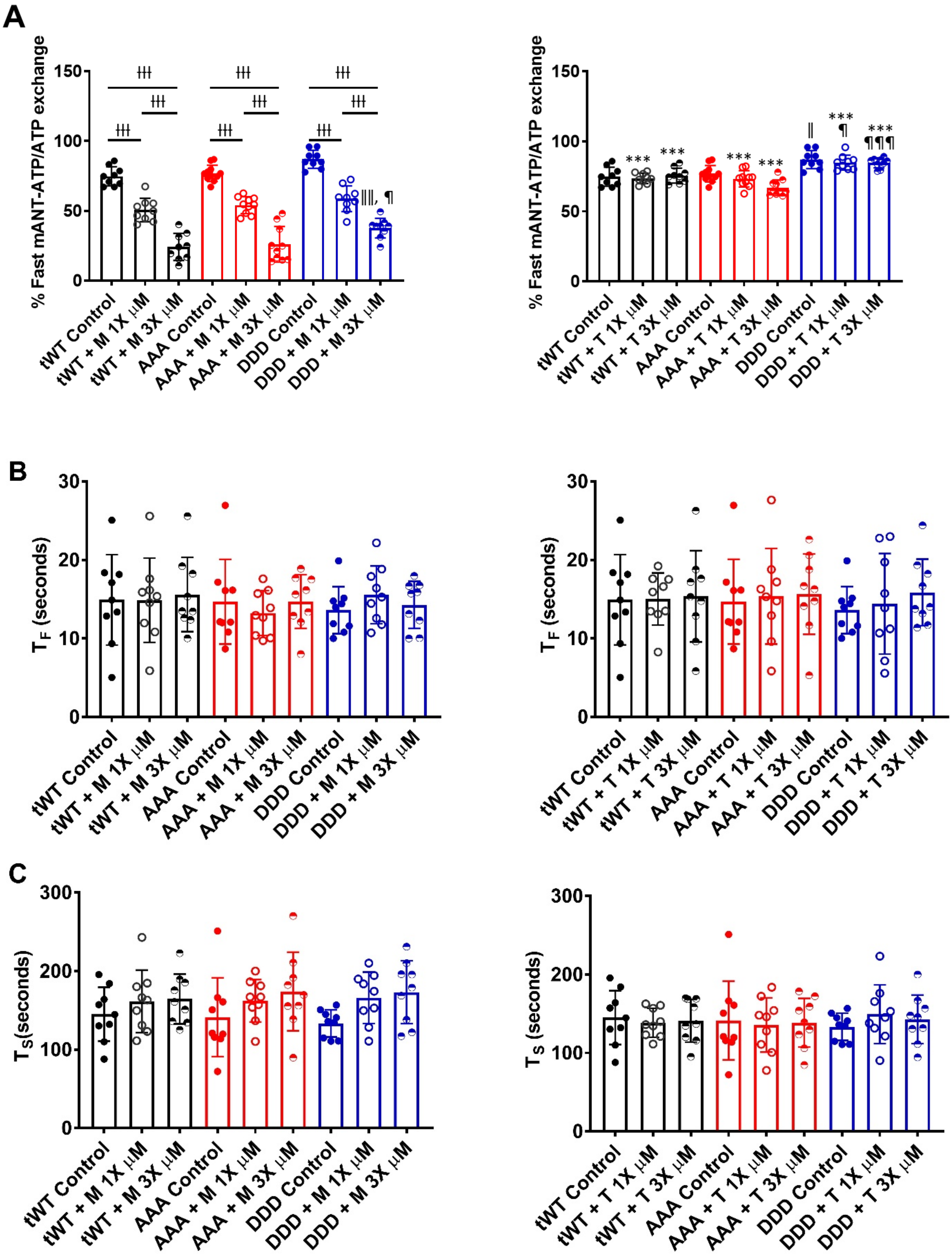
A
% Fast mANT-ATP/ATP exchange
150
100
50
0
ƗƗƗ
ǁǁǁ, ¶
tWT Control
tWT + M 1X µM
tWT + M 3X µM
AAA Control
AAA + M 1X µM
AAA + M 3X µM
DDD Control
DDD + M 1X µM
DDD + M 3X µM
% Fast mANT-ATP/ATP exchange
***
ǁ
¶
¶¶¶
tWT Control
tWT + T 1X µM
tWT + T 3X µM
AAA Control
AAA + T 1X µM
AAA + T 3X µM
DDD Control
DDD + T 1X µM
DDD + T 3X µM
B
T_F (seconds)
30
20
10
0
C
T_S (seconds)
300
200
100
0

Supplemental Figure S6: M decreased the rate of exchange for fluorescent mANT-ATP with ATP in skinned left ventricle muscle fibers compared to T. Bar graphs comparing the fast phase for mANT-ATP exchange (**A**) and lifetimes for the fast (**B**) and slow phases (**C**) across tWt, AAA and DDD muscle fibers in presence of M (left graph) and T (right graph) at 1X (empty circles) and 3X (half-filled circles) concentrations. Refer Table S1 for the values. Statistically significant values were calculated by performing a one-way analysis of variance (ANOVA), Tukey's multiple comparison test (ł, ǂ, * $p<0.05$, łł, ǂǂ, ** $p<0.01$ and łłł, ǂǂǂ, *** $p<0.001$ for 1X vs 3X M, 1X vs 3X T and M vs T at the equivalent dose and transgenic fiber, respectively). N=9-11 fibers per compound treatment in fibers isolated from each transgenic line. Data represented as the group mean ± the standard error mean (SEM). The curves were fitted to custom two-phase exponential decay model.

Supplemental Table S1: Percentage of myosin population for fast turnover of mANT-ATP with ATP and lifetimes for both population of myosin with fast (T1) and slow (T2) turnover of mANT-ATP.

Animal	Compound	Dosage	% of fast myosin population	Lifetime of fast myosin population (T1 secs)	Lifetime of slow myosin population (T2 secs)
tWt	M	Control	74.7 ± 2.1	14.9 ± 1.8	145.04 ± 10.8
		1X	50.5 ± 2.6łłł	14.8 ± 1.6	161.29 ± 12.4
		3X	24.14 ± 3.0łłł‖‖	15.5 ± 1.4	164.50 ± 9.9
	T	Control	73.8± 1.9‖	14.6 ± 1.9	148.43 ± 12.4
		1X	73.5 ± 1.1***	15.01 ± 1.0	138.38 ± 5.78
		3X	75.5 ± 1.6***	15.36 ± 1.8	140.88 ± 8.54
AAA	M	Control	77.69 ± 1.9	14.6 ± 1.6	141.21 ± 15.7
		1X	53.10 ± 1.8łłł	13.2 ± 0.8	162.17 ± 8.4
		3X	24.84 ± 4.0łłł¶	14.7 ± 1.0	173.85 ± 15.7
	T	Control	76.90 ± 2.1	15.2 ± 1.8	143.01 ± 13.6
		1X	73.7 ± 1.9***¶	15.36 ± 1.9	135.67 ± 10.8
		3X	67.06 ± 1.8***¶¶¶	15.64 ± 1.6	138.51 ± 9.7
DDD	M	Control	86.91 ± 2.0	13.6 ± 0.9	133.29 ± 5.4
		1X	58.68 ± 2.9łłł	15.5 ± 1.1	165.97 ± 10.3
		3X	37.6 ± 2.2łłł‖‖¶	14.2 ± 0.9	173.022 ± 12.5
	T	Control	86.26 ± 1.9‖	14.1 ± 1.3	135.12 ± 4.3
		1X	84.86 ± 1.6***¶	14.40 ± 2.0	149.28 ± 11.7
		3X	84.60 ± 1.1***¶¶¶	15.85 ± 1.3	142.77 ± 9.7

Data represented as the group mean ± the standard error mean (SEM). Statistical analyses were performed in all groups using a one-way analysis of variance (ANOVA), Tukey's multiple comparison test with single pooled variance. Five left ventricle fibers were analyzed per animal per compound concentration. * p<0.05, ** p<0.01 and *** p<0.001 M vs T for the equivalent dose; ł, ǂ p<0.05, łł, ǂǂ p<0.01 and łłł, ǂǂǂ p<0.001 for control vs 1X and 3X M and control vs 1X and 3X T, respectively; ‖ p<0.5, ‖‖ p<0.01, ‖‖‖ p<0.001 tWt vs DDD for the equivalent dose; ¶ p<0.05, ¶¶ p<0.01, ¶¶¶ p<0.001 untreated AAA vs DDD for the equivalent dose.
